# Rapid Material Appearance Acquisition Using Consumer Hardware

**DOI:** 10.3390/s141019785

**Published:** 2014-10-22

**Authors:** Jiří Filip, Radomír Vávra, Mikuláš Krupička

**Affiliations:** Institute of Information Theory and Automation of the ASCR, Prague 182 08 , Czech Republic; E-Mails: vavra@utia.cas.cz (R.V.); krupimik@utia.cas.cz (M.K.)

**Keywords:** measurement setup, material appearance, BTF, ABRDF, visual psychophysics

## Abstract

A photo-realistic representation of material appearance can be achieved by means of bidirectional texture function (BTF) capturing a material’s appearance for varying illumination, viewing directions, and spatial pixel coordinates. BTF captures many non-local effects in material structure such as inter-reflections, occlusions, shadowing, or scattering. The acquisition of BTF data is usually time and resource-intensive due to the high dimensionality of BTF data. This results in expensive, complex measurement setups and/or excessively long measurement times. We propose an approximate BTF acquisition setup based on a simple, affordable mechanical gantry containing a consumer camera and two LED lights. It captures a very limited subset of material surface images by shooting several video sequences. A psychophysical study comparing captured and reconstructed data with the reference BTFs of seven tested materials revealed that results of our method show a promising visual quality. Speed of the setup has been demonstrated on measurement of human skin and measurement and modeling of a glue dessication time-varying process. As it allows for fast, inexpensive, acquisition of approximate BTFs, this method can be beneficial to visualization applications demanding less accuracy, where BTF utilization has previously been limited.

## 1. Introduction

**Figure 1.**
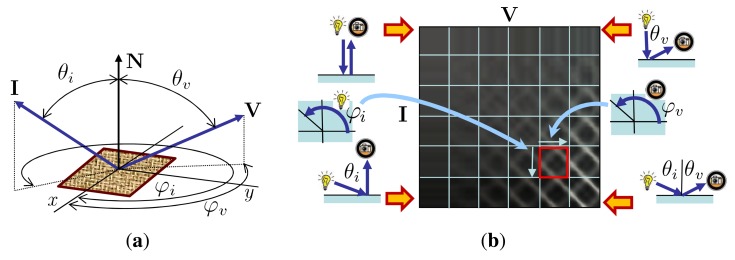
(**a**) A BTF parameterization over material surface; (**b**) a BTF pixel’s apparent BRDF unwrap into a 2D image.

Reproduction of the appearance of real-world materials in virtual environments has been one of the ultimate challenges of computer graphics. Therefore, methods of material appearance representation, acquisition, and rendering have already received a lot of attention. The required material representations depend on the complexity of the material’s appearance. They start with a *bidirectional reflectance distribution function* (BRDF) [1] describing distribution of energy reflected in the viewing direction when illuminated from a specific direction. As the BRDF cannot capture a material’s spatial structure, it has been extended to *spatially-varying BRDF* (SVBRDF) describing the material’s surface appearance by means of a collection of independent BRDFs. This representation allows an already quite efficient approximation of material appearance, mostly based on a wide range of analytical BRDF models. However, the BRDF’s constraints—mainly light and view direction reciprocity—limits the applicability of SVBRDFs to nearly flat opaque surfaces. On the contrary, the *bidirectional texture function* (BTF) [2] does not share these restrictions due to simultaneous measurement of non-local effects in rough material structures, such sub-surface scattering, or inter-reflections. A monospectral BTF is a six-dimensional function *BTF* (*x, y, θ_i_, φ_i_, θ_υ_, φ_υ_*) representing the appearance of a material sample as a surface point with coordinates (*x, y*) for variable illumination **I**(*θ_i_, φ_i_*) and view **V**(*θ_υ_, φ_υ_*) directions, where *θ* and *φ* azimuthal angles, respectively, as shown in Figure 1a.

As the BTF data achieves photo-realistic representation of material appearances without the need for lengthy fitting or tweaking of parameters, it has high application potential mainly in areas requiring physically correct visualizations ranging from computer-aided interior design, visual safety simulations and medical visualizations in dermatology, to digitization of cultural heritage objects. The measurement of BTF is, due to its high dimensionality, very time- and storage-space-demanding. While the storage space issue cannot be easily resolved at the measurement stage and the data are always subject to compression and modeling [3], the duration of the measurement largely depends on the measurement setup design as well as on the types of the sensors and illumination used. To the best of our knowledge, a majority of the current BTF acquisition setups (except [4]) are based on either expensive hardware or specialized equipment demanding laboratory assembly and calibration. As such setups are usually composed of research and custom build devices, the resulting measured data will reflect their high development and purchase costs. This consequently limits the number of publicly available BTF samples as well as their usage in real applications.

**Contribution of the paper:** The main contribution of this paper is a practically verified and (1) technically simple setup and (2) its application for acquisition and reconstruction of approximate BTF from a planar material sample. It allows for rapid acquisition of a BTF subset in six minutes and the fully automatic reconstruction of the entire approximate BTF dataset in under one hour using an inexpensive measurement setup based on an affordable mechanical gantry, a consumer camera and LED lighting. Our setup does not impose any additional restrictions on the measured type of material or its properties (namely in terms of isotropy, reciprocity, bilateral symmetry, opacity), when compared to other BTF setups. We have psychophysically compared our results on seven materials with their ground-true BTF measurements. Moreover, we discuss our method’s limitations, thoroughly analyze its properties, and discuss trade-off between visual accuracy and measurement setup speed/price. Finally, we show setup’s application to human skin measurement and to photo-realistic visualization of time-varying processes.

The paper is structured as follows. Section 2 sets the work in the context of previous work. Section 3 explains the principle of data acquisition and reconstruction. Section 4 describes measurement and data processing procedures, while Section 5 shows results of the proposed acquisition setup. Possible extensions of our work are analyzed and discussed in Section 6, and its applications are shown in Section 7.

## 2. Prior Work

The proposed work relates to methods of SVBRDF and BTF acquisition and their reconstruction from sparse measurements.

As the SVBRDF is restricted by its definition to opaque and almost flat surfaces, its acquisition techniques make use of BRDF reciprocity. A severe limitation of a majority of these approaches is that they capture only isotropic SVBRDF, which is hardly the case for most spatially non-homogeneous real-world materials. On the other hand, BTF is a more general material appearance representation. Such data were initially captured by setups based on gonioreflectometers realizing the required four mechanical degrees of freedom (DOF) of camera/light/sample movement, e.g., [2,5–7]. Because the measurement times were too long (often over 10 hours), certain setups were used to reduce the required number of DOF using parabolic mirrors [8], or a kaleidoscope [9]. They allowed the capture of many viewing directions simultaneously, but at the cost of a limited range of surface height or elevation angles.

The measurement time can also be reduced to approximately two hours by simultaneously using multiple lights and sensors [10], at a high financial cost of such a setup. A recent very detailed comparison of goniometer- and dome-based setups was introduced in [11]. A similar light stage originally designed for human face capturing was used by Gu *et al.* [12] for fast measurement of time-varying processes appearance. The measurement takes only 30 s, however, only 16 views are recorded.

Although the BTF measurement is a very time- and resources-demanding task, not many affordable and fast measurement approaches have been proposed so far.

A solution for the rapid capture of BTF has been proposed by Han and Perlin [9]. Their system is comprised of a triangular mirrored tapered tube that reflects a kaleidoscopic mosaic of a material’s surface as positioned beneath the tube. A fixed camera views the kaleidoscopic image where individual triangular areas correspond to the surface as observed from different viewpoints. The sample is illuminated by a digital projector by covering individual triangles (*i.e.*, generating illumination directions) and uses a beam-splitter to share an optical path with the camera. The main advantage of this system is the exclusion of any moving parts and therefore an extremely fast measurement limited only by the acquisition speed of the camera. However, resulting drawbacks are limited spatial resolution (relative to the camera/projector resolution), restricted dynamic range (as a result of using projector as illumination), greater setup expense due to mirror and projector, and a restricted size of measured sample stemming from an adequate setup size. A final limitation can be offset by increasing the setup’s size, therefore, increasing demands on mirrors size and quality, and consequently incurring greater costs. Also, the requirements for geometrical accuracy and calibration of the system may limit its portability.

An existing statistical acquisition approach [4] allows for fast and inexpensive measurement of a BTF; however, it requires a large sample of the regular material having uniform statistical properties, which is cut and positioned in different orientations with respect to the camera to achieve several viewing directions. The requirement of several sample specimens with the same statistical properties limits practical applicability of the approach to only spatially regular samples. Moreover, the need for extraction of the sample from its original environment prevents many portable measurement scenarios.

In contrast to the approaches presented above, the setup presented in this paper allows approximate BTF measurement and reconstruction of a wide range of materials, without restriction on their properties, need of sample extraction from original environments, or resorting to more expensive and/or fragile laboratory solutions. For a pixel-wise BTF reconstruction from our sparse measurements we applied the BRDF acquisition and reconstruction method introduced in [13]. Its extension to adaptive measurement of multiple slices and more general reconstruction has been proposed in [14]. Although [13] also provides results of BTF reconstruction from the proposed sparse representation, a practical BTF acquisition method has been missing. Therefore, this paper’s main contributions over [13] consist of: (1) the extension of setup’s measurement ability to capture approximate BTF instead of BRDF without introducing additional measurement constraints, which required us to solve a number of practical challenges; (2) thorough analysis of the acquisition method’s performance and options for further improvement of BTF reproduction quality. Moreover, we demonstrate the speed of our setup in two practical applications that are difficult to achieve with other BTF capture systems—human skin BTF measurement, and time-varying BTF measurement and modeling of a dynamic process over one hour.

## 3. Sparse Data Acquisition and Interpolation

Each BTF can be viewed as a collection of pixel-wise apparent BRDFs (ABRDFs), which do not follow the BRDF properties due to non-local effects in a material structure like shadowing, masking, *etc.* If we process individual color channels separately, each pixel’s ABRDF can be represented by a four-dimensional function *ABRDF*(*θ_i_, φ_i_, θ_υ_, φ_υ_*). ABRDF is the most general data representation of material reflectance dependent on local illumination **I**(*θ_i_, φ_i_*) and view **V**(*θ_υ_, φ_υ_*) directions. Its typical parameterization by elevation *θ* and azimuthal *φ* angles is shown in Figure 1a. ABRDF can represent dependence of view and illumination directions of a single BTF pixel. A projection of the 4D ABRDF by means of a 2D image is shown in Figure 1b. Note that individual rectangles (an example is shown in red) represent 2D subspaces of a 4D ABRDF at constant elevations (*θ_i_/θ_υ_*). These subspaces are toroidal. That is, data of the highest *φ* ≈ 2*π* are followed by data of the lowest *φ* ≈ 0.

**Figure 2.**
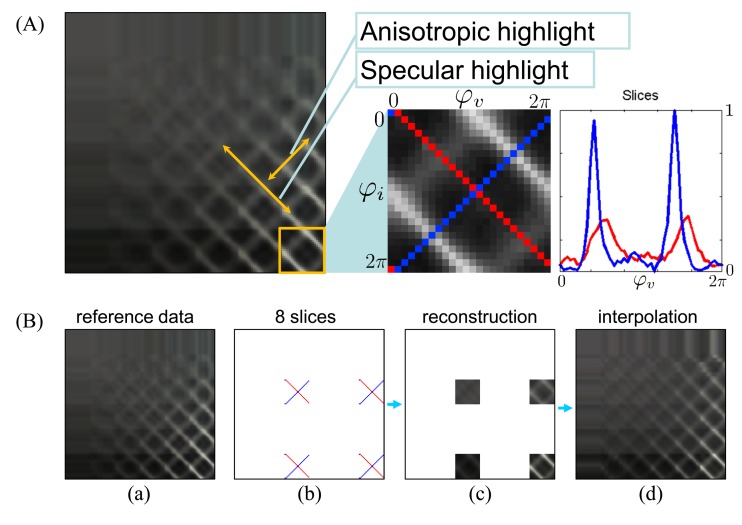
(**A**) A schema of ABRDF subspace representation using two slices and (**B**) principle of entire ABRDF reconstruction from four recorded subspaces: (a) the reference; (b) sparse-sampling of eight slices; (c) reconstructions of elevations where the slices were measured; (d) missing data interpolation.

In this paper, we use ABRDF reconstruction from sparse data proposed in [13]. This method is based on two perpendicular slices measured across azimuthal angles for fixed light and camera elevations as shown in Figure 2A (red and blue). As the slices are perpendicular to anisotropic (the red one) and specular highlights (the blue one) they bear enough information to approximate azimuthally-dependent behavior of the ABRDF.

A principle of the ABRDF reconstruction method is sketched in Figure 2B. First, the sparse samples are measured at predefined locations (azimuthal angles) for defined elevations. Then four sampled ABRDF toroidal subspaces are reconstructed from the values of the slices, and finally the remaining values are interpolated. All the BTF pixels, *i.e.*, ABRDFs are processed separately in this way.

## 4. Approximate BTF Measurement

This Section describes a fast, practical measurement setup of capturing the slices in BTF space using a consumer camera and a LED point-light source, followed by a complete BTF dataset reconstruction from such measurements.

### 4.1. Acquisition Setup

**Figure 3.**
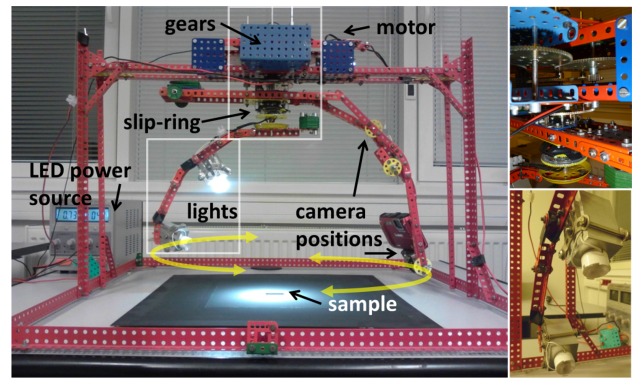
The proposed BTF measurement device.

The setup realizing measurement of the BTF slices is shown in Figure 3. It consists of a mechanical gantry holding two arms rotating synchronously in either the same or opposite direction. The gantry was built using a Merkur toy construction set (http://www.merkurtoys.cz/en/). Contrary to its initial version presented in [13], requiring manual movement of the arms, we use a more reliable solution with a single DC motor (4.5 V) run at a constant speed. Using additional gears guarantees accurate arms synchronization and allows us to switch the mutual rotation directions of the arms (see Figure 3-top-right). One of the arms holds two LED Cree XLamp XM-L light sources with 20° frosted optics (see Figure 3-bottom-right). Contrary to the original design we improved arms weight balancing and added a rotation slip-ring to avoid clumsy LED power supply (0.7 A/3 V) wires. The second arm has two positions for attachment of a Panasonic Lumix DMC-FT3 camera. One advantage of this camera is that it does not have a protruding lens, which could possibly block the arm bearing the lights. Elevations of the LEDs and camera in both positions are fixed at 30° and 65°. The setup can be constructed in 10 h, for less than $350 and can hold almost any compact camera. The setup’s dimensions are 0.6 × 0.6 × 0.4 m, with a weight of 6 kg. The frame holding the rotating arms can be folded down on the support platform, so the setup’s size can even be reduced to allow easy transportation and the use for field measurements of samples that cannot be removed from their environments.

A material sample is placed below the setup under the arms’ rotation axis (see Figure 3). The axial slice data (red) are measured using rotation of the mutually fixed light and sensor around the sample, while the diagonal slice (blue) data are obtained by mutually opposite movements of the light and sensor with respect to the sample. In both cases, the camera and light travel full circle around the sample and return to their initial positions.

### 4.2. Sparse BTF Data Capturing

The camera records the material sample’s appearance at different arm positions as a video sequence at a resolution of 1280 × 720 pixels. Both *s_A_* and *s_D_* slices are recorded for two different elevations of the camera (C1, C2) and light (L1, L2); therefore, eight slices are measured at approximate elevations *θ_i_/θ_υ_* = [30°/30°, 30°/65°, 65°/30°, 65°/65°] as shown in Figure 2B(b). Recording of a single slice takes 30 s and the entire set of eight slices is captured in six minutes—during which time the LEDs are switched four times, gears twice, and camera position once. Note that the fully automatic device assembled with two cameras would need only two minutes. Camera zoom and white balance are fixed during the recording to allow the calibrations described in Section 4.3. The camera’s image stabilizer was switched on and the global exposure level was set to $-\frac{2}{3}\text{EV}$ to avoid overexposure. Note that, although the exposure level of individual measured images is unknown, the global exposure level adjustment by an uniform change of exposure time/aperture can be used to shift dynamic range of the whole recorded image sequence.

**Figure 4.**
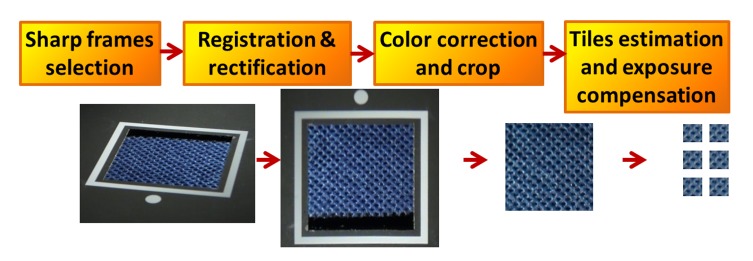
The pipeline of each slice’s frame processing.

Due to a short distance of the camera and LED lights from the sample, we have limited the size of the measured sample to 30 × 30 mm, which is sufficient for a wide range of materials. However, shadows cast by the registration frame (see Figure 4) limits the effective size to ≈ 80%. Although a larger sample size can be used, we limited it due to a large span of illumination and view angles across sample’s plane, possibly causing spatial reflectance non-uniformity in the captured images. Finally, we cut the smallest possible repetitive tiles found near the sample’s image center. A white border with a white dot was attached around the sample (see Figure 4) for detection of the camera orientation with respect to the sample, and for frame registration.

### 4.3. Data Processing and Calibrations

Basic processing steps applied to the measured data are outlined in Figure 4 and explained in more detail in this Section.

**Frame Filtering and Registration:** Eight slices are recorded as videos at a frame rate of 30 fps, *i.e.*, 900 frames per slice. As the camera provides M-JPEG non-interleaved variable bit-rate format storing all recorded frames (*i.e.*, not only the key-frames), the error introduced by video coding is negligible compared to the ABRDF reconstruction error. From each of the eight video sequences, 24 frames are extracted corresponding to the sampling of azimuthal angles *φ_i_/φ_υ_* every 15°. However, as not all of the frames are sharp due to motion blur we search in the neighborhood of ± 4 frames for the sharpest image minimizing the edge-based blur metric [15]. As this approach might miss intensity at specular reflections, we additionally scan the entire sequence for the two brightest frames, which are always sampled. To capture even very sharp specular highlights, four additional samples 1° apart from specular reflections are recorded as well. This leaves us with a set of 28 frames for each diagonal slice (perpendicular to specular highlights; shown in blue in Figure 2A), and 24 frames for each axial slice (shown in red), *i.e.*, 208 frames in total. Note, that in the practical measurement of diagonal slice 0–2 frames are removed due to the occlusion of the camera view by lights; therefore, the number of frames eventually selected is slightly lower. A subset of the recorded frames is used for camera calibration (http://www.vision.caltech.edu/bouguetj/calib_doc/) and further for geometric distortion compensation of all frames [16]. The frame registration itself is performed in two steps. First, all images are registered based on the sample’s border line detection using the Hough transform and computing homography between their intersections and desired corner coordinates. Second, as the measured material sample’s plane is usually at least 0.1 mm below the registration plane defined by the white border, we detect and compensate for this height and angular misalignment using the iterative fitting method [17]. This method uses PCA-based image compression as the alignment quality measure. Finally, the registered images are cropped to a size of 300 × 300 pixels, yielding a resolution of 340 DPI. If the sequences were recorded in a Full HD resolution (1900 × 1080 pixels) the BTF resolution would approach 500 DPI.

**Exposure and Light Non-Uniformity Compensation:** Unfortunately, most compact cameras adapt their exposure depending on the amount of light coming from the scene, which is also true for the camera we used. On a positive note, this enabled us to capture as much information as possible, even using a limited dynamic range of the camera’s sensor (8 bits/channel). However, the information about exposure throughout the video sequence could not be retrieved from an EXIF header as is possible for still photos. Another related problem is the spatial non-uniformity of illumination, which is caused by a limited distance of LEDs from the sample and the fact that we use point-light instead of directional illumination. Therefore, we compensate for the exposure fluctuations as well as for the spatial non-uniformity of illumination of the original images *I* using the intensity of black uniform material with known BRDF at the locations beyond the white border surrounding the measured sample (Figure 4). A compensation image *C* is computed for each frame by linear interpolation of the black material intensity. First, the originally measured frames in slices *I* are resampled to the azimuthally uniform grid *I_G_*, which step is necessary for ABRDF subspace reconstruction. Pixels of the monospectral correction image *K* represent value in the center of the image divided by value interpolated from measured intensities behind the white frame, *i.e.*, this value includes the known reference BRDF of the black material *B* divided by the compensation image *C* for the corresponding angles

(1) $$K_{i}(x,y)=\frac{1}{3}\overset{3}{\underset{j=1}{\text{∑}}}\frac{B(\xi (i),j)}{C_{i}(x,y,j)}$$

where *i* = 1… *m* is the number of the frame in the slice of size *m, j* is a color channel and *ξ* is a known mapping function *ξ*(*i*) → (*θ_i_, φ_i_, θ_υ_, φ_υ_*).

The compensated image *I_C_* is obtained as

(2) $$I_{C,i}(x,y,j)=I_{G,i}(x,y,j)K_{i}(x,y)$$

Reference BRDFs of the black target *B* were obtained from the UTIA BTF database (http://btf.utia.cas.cz).

**Obtaining View and Illumination Directions:** When all frames have been registered and compensated, their corresponding illumination and viewing angles must be identified. The camera viewing angles *θ_υ_/φ_υ_* are obtained from the camera’s extrinsic parameters, given the known camera calibration and corner points of the sample’s borders. Coordinates of these points are obtained from the image registration based on the camera calibration. As the viewing angles are known and the lighting support arm is mechanically coupled with the camera support arm (Figure 3), the illumination azimuth angle can be computed as: *φ_i_* = *φ_υ_* + *α* for the axial slice *s_A_*, and *φ_i_* = 2*π* + *α* − *φ_υ_* for the diagonal slice *s_D_*. The elevation angles *θ_i_* are estimated from the fixed vertical positions of the LEDs in the setup (Figure 3).

**Colorimetric Calibration:** The compensated images are further colorimetrically compensated using a transformation matrix relating measured and known color values of the ColorGauge Micro Target (35 × 41 mm) in the least-square sense [18]. This target has colors of the same specification as the X-Rite ColorChecker Classic target, however, its physical size is reduced.

**Entire BTF Space Reconstruction:** Since direct use of material images for BTF rendering would exhibit distractive seams on textured surfaces, we employ an image-tiling approach to find seamless tiles [19]. Finally, the compensated and tiled images of the material’s appearance for the known illumination and viewing directions (measured in slices) are used for reconstructing the remaining directions. Due to a lower computation complexity we used step-wise linear interpolation of the entire illumination and view space instead of the more accurate but substantially slower global interpolation approach as shown in [13].

**Timings:** A typical timeframe for data processing using a desktop PC with Intel i7-3610QM 2.3 GHz is as follows: video sequences decoding, 1 min; obtaining sharp frames, 15 min; image registration and registration plane alignment, 15 min; image-tiling, 5 min—in total 36 min. As the reconstruction of a single BTF pixel from the slices takes 0.2 s, the reconstruction of a BTF tile of the size 128 × 128 takes under one hour using a single core or 17 min using four cores. To summarize, our method allows measurement and reconstruction of BTF in under one hour.

## 5. Results

We used seven BTF samples from the UTIA BTF database (http://btf.utia.cas.cz) for evaluation of our method. These measurements cover a hemisphere of illuminations/views in 81 directions [6]. One advantage of this database is that it provides physical specimens of some of the measured samples for research purposes. Therefore, we can directly compare measured reference BTF data with their approximation as captured by our acquisition setup.

**Figure 5.**
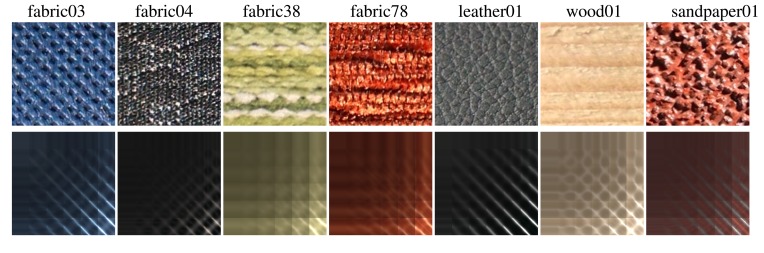
The measured materials on an area of 15 × 15 mm (the first row) and their average ABRDFs (the second row).

We selected different types of materials for measurement as illustrated in Figure 5: non-woven fabric (*fabric03*), upholstery with a height profile (*fabric04*), woven fabric (*fabric38*), and corduroy-like upholstery (*fabric78*), artificial leather (*leather01*), raw wood (*wood01*), and rough sandpaper (*sandpaper01*), most of them highly anisotropic as is clear from their spatially averaged ABRDFs shown in the second row.

As the reference measurements have a higher resolution (353 or 1071 DPI) than the data resolution captured by our camera (340 DPI), we downsampled the reference data to match our lower resolution. We also attempted to cut similar tiles [19] from both the reference data and the captured BTF datasets to achieve a fair comparison of our measurements with the reference BTF data.

**Figure 6.**
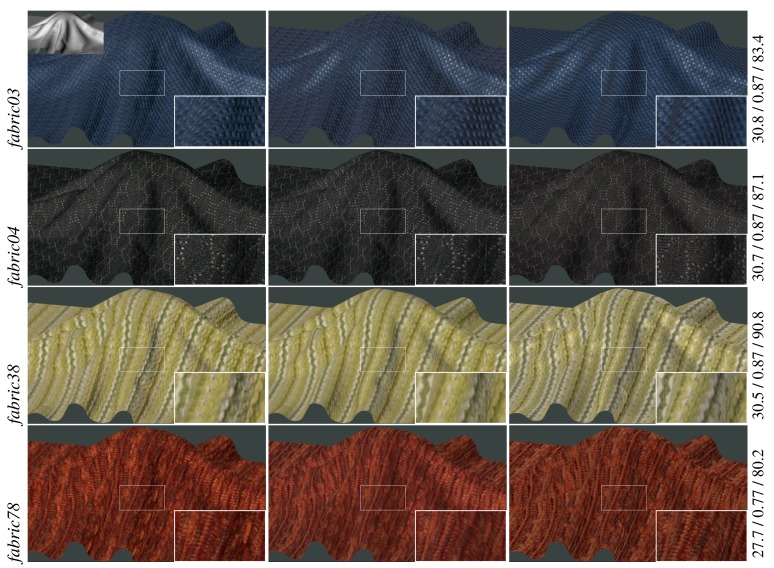
A comparison of renderings: (left) using the entire reference BTF datasets (6561 images), (middle) using BTF reconstruction from a sparse subset of reference BTF (208 images), (right) using BTF reconstruction of the proposed measurements (208 images). At the end of each row are PSNR[dB]/SSIM/VDP2 values between the first two images.

**Figure 7.**
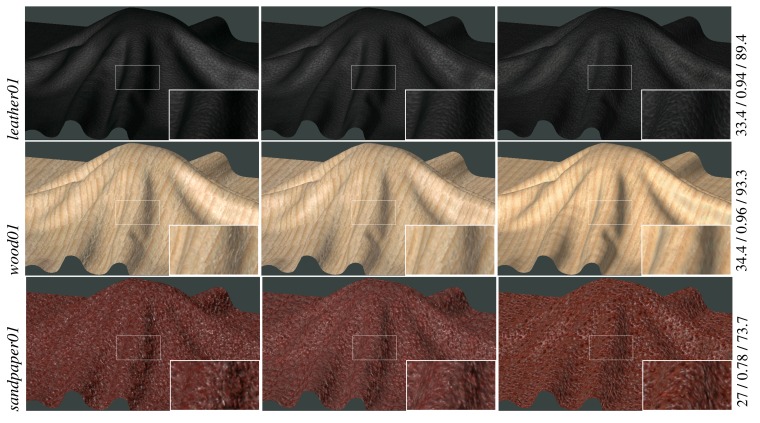
A comparison of renderings: (left) using the entire reference BTF datasets (6561 images); (middle) using BTF reconstruction from a sparse subset of reference BTF (208 images); (right) using BTF reconstruction of the proposed measurements (208 images). At the end of each row are PSNR[dB]/SSIM/VDP2 values between the first two images.

A comparison of the reference BTF data with our sparsely measured and reconstructed BTF datasets is shown in Figure 6 (fabrics) and Figure 7 (leather, wood, sandpaper). For the purpose of distinguishing between differences introduced by the reconstruction procedure and those resulting from the proposed acquisition technique, the reference measurements (the left column) are compared with two types of results. The first one (the middle column) reconstructs BTF from the subset of *reference* measurements (208 images) while the second one (the right column) reconstructs BTF from the same subset of images, however, measured by the *proposed acquisition setup*.

These images show a close resemblance of both results to the reference data. The main difference between them is in color hue caused by differences in the acquisition processes as discussed in Section 6. Smoother appearance of the material *wood01* results from variations in the amount of sanding that has been performed on the raw wood surface. Note that, although we use the same materials as the reference measurement system, we cannot achieve pixel-wise alignment between the reference and the proposed measurements. Therefore, the accuracy of the proposed method cannot be assessed by any of the standard pixel-wise quality evaluation metrics. This type of comparison is possible only between data in the first two columns. The difference values for PSNR[dB], SSIM, and VDP2 are shown on the right sides of the images.

### 5.1. Perceptual Evaluation

To objectively evaluate our measurement method, we ran two psychophysical experiments. In the first one, we evaluated visual error introduced by means of sparse sampling as compared to complete BTF sampling. The second experiment compared the performance of reference and proposed setups recording the same sparse set of images; in other words, a visual quality trade-off occurred by using the proposed instead of the reference setup.

**Figure 8.**
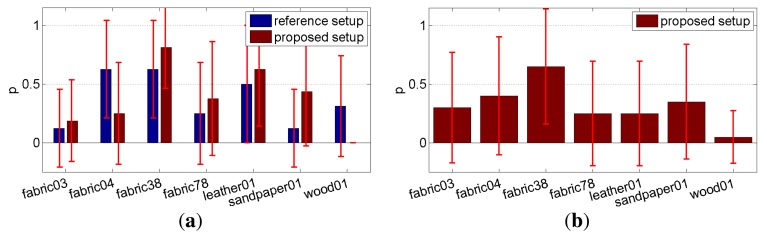
Results of psychophysical experiments: (**a**) *Experiment 1* – comparing results of sparse measurement methods *vs.* full reference BTF data as a perceived probability *p* of sparse data; (**b**) *Experiment 2* – comparing quality of the sparse measurement setups as a perceived probability *p* of data from the proposed setup.

*Experiment 1* – Eight naive subjects compared difference between reference and sparsely sampled BTF representations. Subjects were shown two rendered images on a calibrated screen together with actual specimens of materials as shown in Figure 5. The question was: *Which of the two images looks more realistic?* When we compared the renderings of complete and sparse reference data (the first *vs.* second column in Figures 6 and 7), on average, 37% of our subjects preferred those from the sparse reference data, and when we compared renderings of complete reference data with our sparse measurements (the first *vs.* third columns in Figures 6 and 7), 38% of our subjects preferred those from our data. The results for individual materials are shown in Figure 8a. Interestingly, for materials *fabric38, fabric78*, and *sandpaper01* scored the proposed measurement method much better than sparse reference one. Note that the difference for material *wood01* is a result of a slightly different specimen of the same material, with a smoother surface finishing.

**Figure 9.**
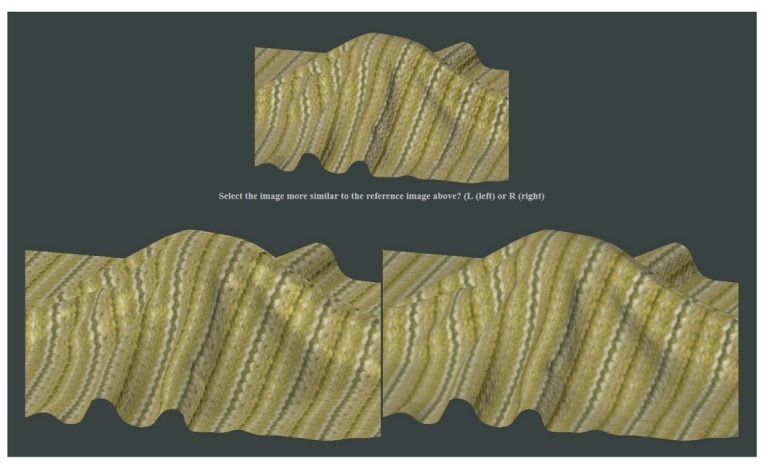
Example stimulus image from the second psychophysical experiment.

*Experiment 2* –To determine error resulting from the proposed simplified acquisition technique when compared to sparse reference measurement, we ran another perceptual study with 20 subjects. For each tested material they were shown reference measurements rendering using a complete set of 6561 BTF images and were asked to choose between the two sparse measurement options using 208 images only (obtained using the proposed and reference setups). An example stimulus image is shown in Figure 9; subjects were asked: *Select the image more similar to the reference image above*. We used a website-based interface, and image couples were ordered randomly. Subjects were encouraged to consider a overall appearance instead of focusing on local details. To avoid the latter we reduced size of the reference image. Analysis of responses revealed that on average, only 32% of subjects considered the results from the proposed setup more similar to the complete reference data (see Figure 8b), while 68% of subjects preferred data from the reference setup. When material *wood01* is excluded (as explained above) the ratio becomes 38%:62%. Although the lower value likely resulted due to similarity of the complete reference dataset to its sparse subset (both sharing the same acquisition process, *i.e.*, color hue, texture tiles), the result is encouraging, especially when considering the relatively high standard deviation of subject responses shown as errorbars in Figure 8b.

In summation, although results invariably depend on the material being measured, we found that the average subject was able to recognize differences between complete and sparse measurements as well as distinction between data from reference and proposed setups. However, high standard deviations in both experiments suggest that feedback significantly depends on personal preference rather than unequivocal visual differences resulting from using the simplified measurement setup. Therefore, we believe, that even though the reconstruction from sparse samples is not always physically accurate (in terms of properly shading of structural elements), the performed perceptual study confirmed our setup captures the overall look-and-feel of a given material’s appearance.

## 6. Discussion

### 6.1. Advantages

A notable advantage of our setup is its ability to quickly (≈ 5 min) measure almost any flat and slightly rough materials without needing to extract them from their environment. Compared to the SVBRDF measurement and representation approaches, the proposed method is not limited to the restrictions imposed by the BRDF properties. Therefore, it can be found useful for fast and inexpensive approximate BTF acquisition of many materials. Any height difference between the material surface and the registration plane is compensated for and the entire BTF is reconstructed in under one hour. Therefore, our method can be used for fast and inexpensive measurements of such samples as human skin (see Section 7), precious planar cultural heritage artifacts, e.g., coins, engravings, fabrics, *etc.*

### 6.2. Limitations

To achieve fast and portable appearance measurements, our method restricts the number of measured images to 208 and this fact is reflected in certain limitations of its visual accuracy. Although there are not any restrictions imposed on the measured materials when compared with other BTF capture setups, the results have shown the following general limitations:

- *Lower sharpness of structure details* – results from geometrical deformation of the structure’s features, which is caused partly by mechanical vibrations during the measurement and partly by very sparse sampling of the azimuthal space, as well as by interpolation of the data at missing elevations. The interpolation causes blurring due to improper highlight extrapolation for low elevation angles. Another reason for the lower contrast is a low dynamic range of the camera sensor, where certain details are lost after the exposure compensation (e.g., white dots in *fabric04*); therefore, a sensor with a dynamic range over 8 bits/channel would help.
- *Color hue differences* – are due to different dynamic range and spectral response of the RGB sensors used for the reference and our data acquisition, and due to different calibration targets used. Another source of these differences can be slight color variations across the specimen plane (e.g., *sandpaper01*).
- *Visible repeatable seams* – are caused by tiling with the aid of only a single BTF tile and by less than ideal illumination non-uniformity compensation, and are apparent for samples represented by a very large tile.
- *Limited sample size* – A larger sample size is not a severe limitation of our setup, as larger areas can be, due to a high speed of the measurement, scanned sequentially; and this approach does not compromise the setup portability. Alternatively, the setup can be built in a larger size for only minor additional costs.
- *Highly specular samples* – could be inappropriately represented using the proposed fixed-sampling approach and would require adaptive sampling based either on initial material scan or on a step-wise adaptive approach [14].

### 6.3. Data Reconstruction Performance Analysis

**Figure 10.**
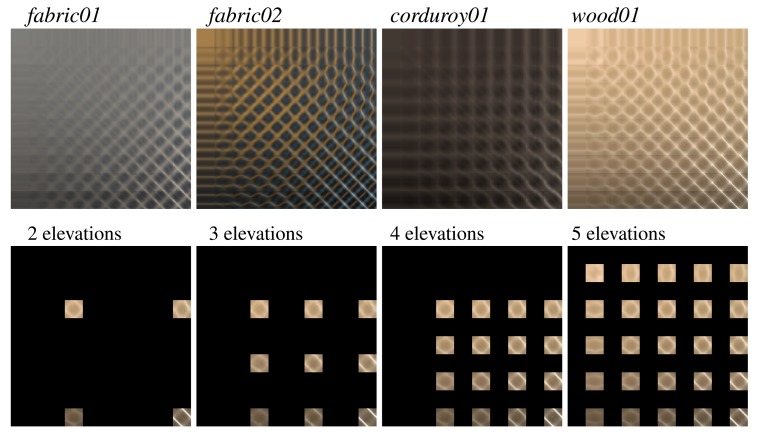
Tested BRDFs of fabrics and wood (**top**), and example of sampled BRDF subspaces at four different tested sets of elevations (**bottom**).

As the ABRDF data reconstruction is the main source of error in applications of our setup, we analyzed influence of: (a) number of elevations used for ABRDF measurement and (b) number of slices used for subspace reconstruction. In our analysis we used four ABRDFs of highly anisotropic materials from the UTIA BTF Database. Each material was measured as BTF with ten elevations (7.5° apart) and 24 × 24 azimuthal samples per subspace. Finally, all pixel values were averaged (see Figure 10-top). Note that elevation angles of these measurements are different from those used in the proposed setup; however, we believe that the following analysis will bring to light information on the reconstruction power of the acquisition method and its consequences for possible extension and the scalability of the proposed setup.

We analyzed reconstruction performance for four different combinations of elevations where the subspaces were measured: two (30° and 75°, used in this paper), three (30°, 52.5°, 75°), four (30°, 45°, 60°, 75°), and five (15°, 30°, 45°, 60°, 75°) as shown in Figure 10-bottom.

**Figure 11.**
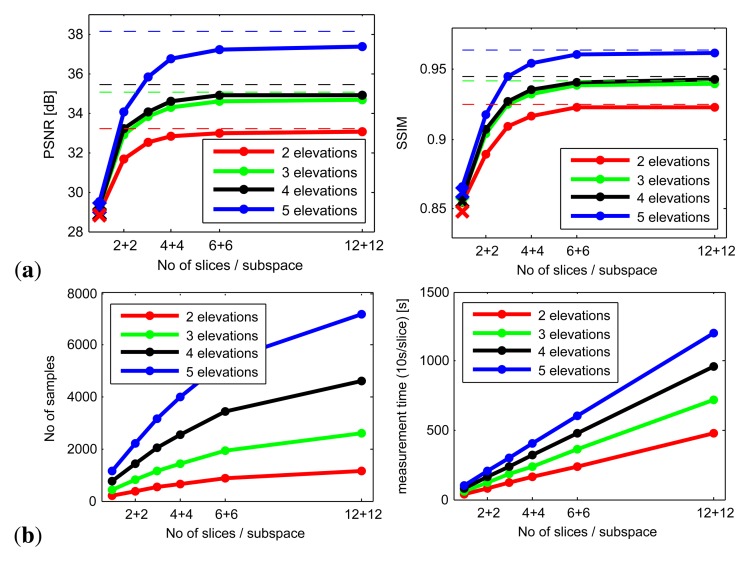
(**a**) Reconstruction error of entire BRDF left: PSNR, right: SSIM); (**b**) a total number of samples (left) and measurement time (right) as a functions of a number of sampled elevations and slices per subspace.

This reconstruction can be performed either from only two slices by means of the original method proposed in [13] or from an arbitrary number of slices by means of a recent, more general interpolation approach [14]. Our analysis revealed that the main quality bottleneck resides in subspace reconstruction using only two slices. Therefore, significant improvement can be achieved by increasing the number of measured slices. Figure 11a) shows PSNR and SSIM errors of the reconstruction method [14] averaged across the tested ABRDFs for linearly increasing number of slices, while the dashed lines represent lower error bounds of the subspace sampling (using all 24 × 24 samples/subspace). The biggest difference from reference was obtained for material *wood01*, where the sharp highlights could not be retrieved by means of the coarse sampling of the data using azimuthal step 15 degrees. Note that this is not a principal disadvantage of the proposed setup, as the sampling along the slices can be arbitrarily dense or even adaptive [14]. Similar performance was observed also for MSE and VDP2 metrics. Already 4 axial and 4 diagonal (4 + 4) slices uniformly distributed across subspace deliver a reasonably low ABRDF reconstruction error, compared to mere 1 + 1 slices (reconstruction using method [13] is shown as crosses). The reconstruction error also depends on the density of subspace sampling across elevations (represented by charts of different colors) where the quality gains start to be significant from subspace sampling using more than 3 + 3 slices. The main quality contribution is gained using additional middle elevation between 30° and 75° and first of all additional low elevation 15°. We have also found that using only four elevations (15°, 30°, 52.5°, 75°) instead of five (15°, 30°, 45°, 60°, 75°) provides reconstruction very similar to the five tested elevations (a difference of less than 0.5%; not shown in graphs).

Graphs in Figure 11b mark an almost linear increase of measured samples count and time for measurement with number of slices recorded per subspace. For instance, four cameras can record material appearance for each light in parallel and the measurement time for 8 slices/subspace (e.g., 4 + 4, 2 + 6) can be around 7 min. Note that this time assumes arms positioned overhead and measurement speed of 10 s/slice, that we believe can be achieved with a steadier gantry and sufficient illumination intensity.

Finally, we analyzed a configuration of our setup comparable with a standard BTF capture system in terms of measurement *vs.* number of images taken. The fastest BTF measurement setup [10] collects and processes 151 × 151 = 22801 images within two hours. Our approach using 10 cameras and lights could measure 1600 slices (assuming speed 10 s/slice) within 30 min and process them in the remaining 90 min. Total 1600 slices at ten view and ten illumination elevations result in 16 slices per subspace, e.g., a configuration of 8 + 8 slices. The azimuthal density is restricted only by the video frame rate, e.g., for a step of 15° it is 24 samples per slice, and we obtain 38,400 samples. If we were to use a steadier gantry, the searching of sharp frames could be avoided and we could achieve a processing time of ≈30 s/slice; *i.e.*, allowing BTF reconstruction in 90 min using a PC with 8 cores. Alternatively, slices processing can be partly done during the measurement process provided the cameras allow for direct streaming of the captured data to a PC. When we compared the reference ABRDFs measured at angular resolution 151 × 151 with the simulated measurement and reconstruction using the proposed configuration, we obtained PSNR 50.1 (averaged across all four materials). Similarly for 4 + 4 (19,199 samples) and 4 + 6 (24,209 samples) slices per subspace we obtain PSNR 44.2 and 46.8 respectively. Although this setup’s configuration would still be less expensive than the setup with 151 cameras [10], we consider it reasonable to limit the number of cameras and slices, allowing a much higher speed of the BTF acquisition while maintaining the required visual quality.

### 6.4. Options for Further Improvement

Our analysis revealed that the reasonable trade-off between visual quality and measurement time can be achieved using four cameras and lights placed at elevations 15°, 30°, 52.5°, 75°, recording at least six slices per subspace. Measurement using this setup configuration would take 7 min. One also must consider the increased price of such a setup requiring four cameras.

We believe that our setup has, due to its scalability, high application potential for development of BTF measurement systems with performance adapted to the requested visual quality or speed. Although different materials require different numbers/distributions of samples, we fixed, the number of samples in our experiments due to the diffuse character of the tested materials. One option for further improvement would be adaptive selection of samples from video (along the slices) based on the overall material reflectance as shown in [14].

## 7. Applications

**Figure 12.**
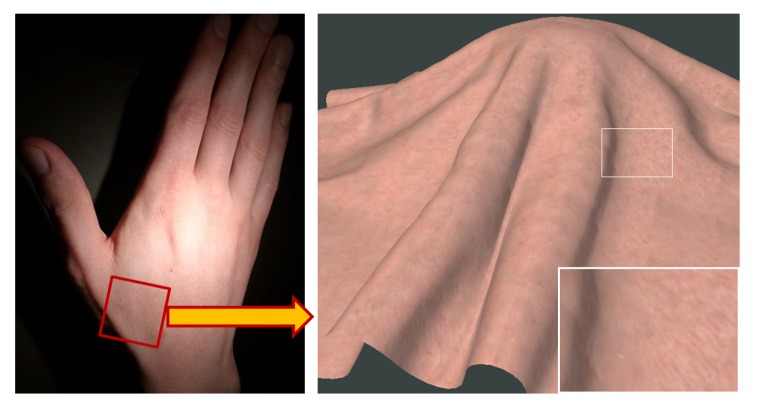
Measured BTF of human skin.

To demonstrate the speed and portability of our acquisition procedure (using 2 illumination and 2 view elevations) we recorded and reconstructed BTF of skin on the author’s hand (Figure 12). Although there is present a certain blur caused by slight movement of subject’s hand during the five minute measurement, note that such a measurement would not be possible, due to long measurement times or a setup design, by any BTF acquisition method presented so far. Compared to [12] our approach is not constrained by the BRDF properties, *i.e.*, we can also record rougher, non-opaque materials.

**Figure 13.**
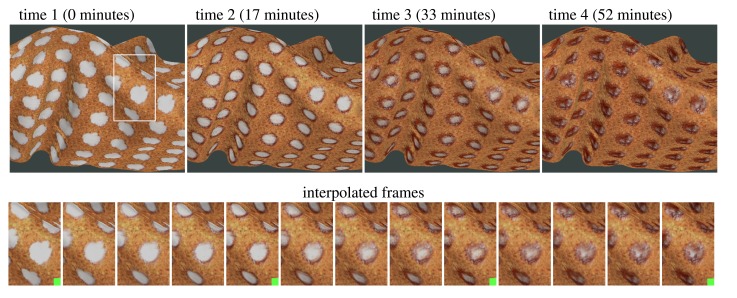
Four measurements of time-varying BTF of glue desiccation (the first row). A complete sequence rendered from interpolated BTFs based on the four measured (marked as green) and nine BTF time-frames interpolated using displacement interpolation (the second row).

The speed of our setup also allows us, contrary to other setups [20], to record time-varying BTF of dynamic processes at a sampling rate of up to 12 measurements/h). We measured desiccation of a glue dot placed on the underside of leather as shown in the first row of Figure 13 (measurements are replicated using an image-tiling approach to show the dot’s illumination- and view-dependent appearance). We performed four measurements, *i.e.*, recorded the BTF of the process every 15 min in the span of one hour. Typical duration of a single measurement was between 4–5 min.

**Figure 14.**
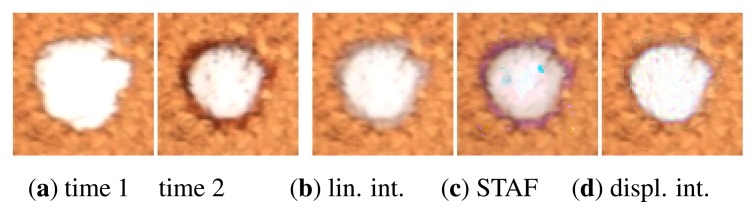
Interpolation of the time-frames 1 and 2 (**a**) (Δt = 17 min, 71 × 70 pixels) using: (**b**) linear interpolation; (**c**) STAF algorithm; and (**d**) displacement interpolation (DI).

Then we tested three interpolation methods: linear interpolation (applied to TVBTF in [20]), space-time factorization (STAF) [12], and displacement interpolation [21] as shown in Figure 14. As expected, the linear interpolation was fastest, but introduced artifact blur effects at the regions where the glue area shrinks. The STAF algorithm performed significantly better, however, it is sensitive to careful setting of initial conditions as it performs per-pixel non-linear fitting. Finally, we tested the displacement interpolation (DI) solving a generalized mass transport optimization problem. In contrast to the STAF algorithm modeling spatial-temporal dynamics of the entire time-sequence, the DI algorithm performs weighted interpolation between only two images. Due to its stability we resorted to a DI algorithm and applied it to interpolation between two sets of 208 images representing two time frames in TVBTF. The second row in Figure 13 shows the obtained smooth transitions between measured time-frames illustrating illumination and view-dependent effect of glue desiccation over one hour (the measured time-frames are marked by green square).

Contrary to the acquisition setup introduced by Gu *et al.* [12], our setup has a slightly longer acquisition time; however, it is based on inexpensive components and provides a higher number of viewing directions.

## 8. Conclusions

We present a fast and inexpensive setup for material appearance acquisition in the form of an approximate bidirectional texture function (BTF). The proposed acquisition setup is based purely on consumer hardware and is easy to build. The data acquisition and subsequent fully automatic reconstruction of the entire BTF dataset is fast and computationally non-intensive. The measurement process records a material’s appearance using eight video sequences, from which only 208 frames are taken to approximate the entire BTF of the measured sample. The promising performance of this method has been thoroughly psychophysically compared with reference BTF measurements. Further analysis has shown that the proposed setup offers sufficient scalability to meet different requirements on acquisition quality and speed. Moreover, we have demonstrated flexibility of the acquisition process on examples of human skin and dynamic processes measurement.

Although the presented setup has certain limitations and does not capture exact detailed appearance for some materials, we believe that its speed, simplicity, and portability will make these approximate BTF measurements accessible even to such applications for which the standard BTF acquisition methods are too expensive.

In our future work we plan to employ material-dependent sampling along the measured slices and render material appearance on GPU directly from the sparsely measured dataset without the need for reconstructing a complete BTF dataset.
